# Scaling the Analytical Information Given by Several Types of Colorimetric and Spectroscopic Instruments Including Smartphones: Rules for Their Use and Establishing Figures of Merit of Solid Chemosensors

**DOI:** 10.1021/acs.analchem.0c03994

**Published:** 2021-04-05

**Authors:** Adria Martínez-Aviño, Carmen Molins-Legua, Campíns-Falcó Pilar

**Affiliations:** MINTOTA Research Group, Departament de Química Analítica, Facultat de Química, Universitat de València, Dr. Moliner 50, 46100 Burjassot, Valencia, Spain

## Abstract

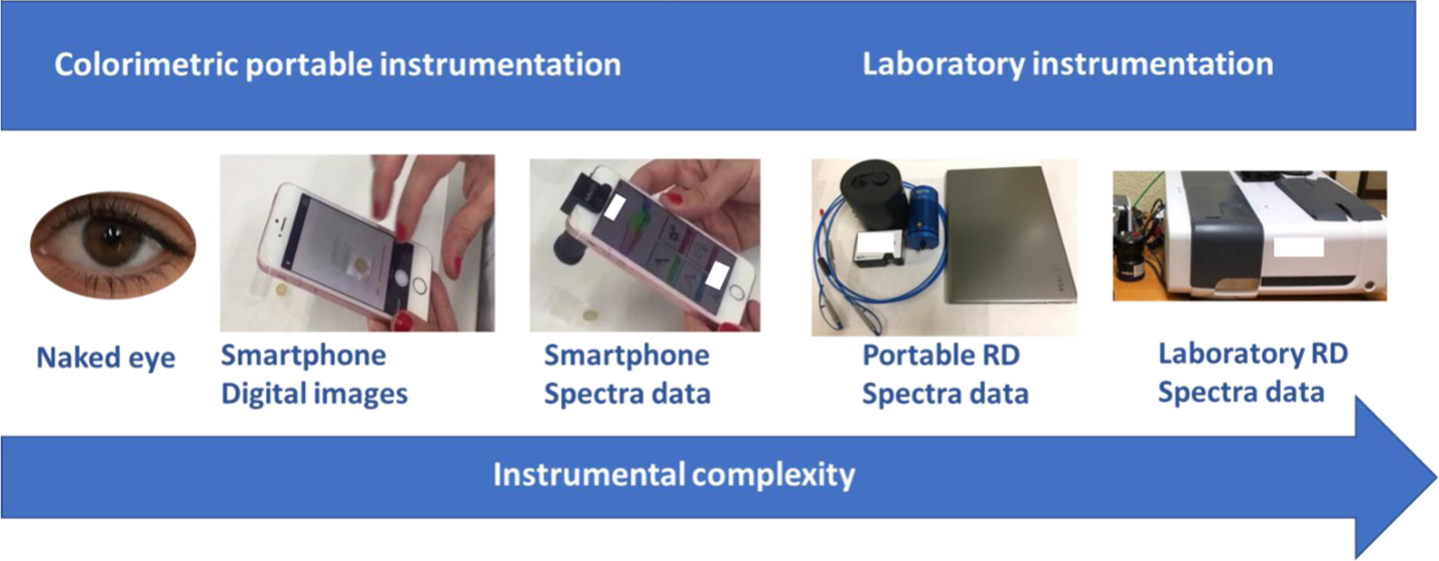

The analytical information given by different types of instruments was scaled in order to establish suitably the figures of merit of a given methodology based on color measurements. Different lab and portable instruments, including smartphones with and without a miniaturized spectrophotometer accessory, have been tested. In order to obtain broad information and using objective criteria, these instruments have been compared from (1) the analytical point of view, considering mainly the detection limit (limits of detection [LODs]), selectivity, accuracy and intra- and interday precision, size, components, and costs; and (2) the environmental point of view, based on their footprint as kilograms of CO_2_. No significant differences in the precision were obtained with RSD (%) values lower than 10% for all of the instruments, but the achieved values of LOD, selectivity, accuracy, and cost were different. Footprints of CO_2_ were better for portable instrumentation, especially for smartphones. Three solid chemosensors made of different materials (PDMS, paper, or nylon) have been tested for the determination of ammonia and hydrogen sulfide at different concentration levels (ppb levels). As a result of this study, some rules for selecting the instrument for obtaining the required information have been established. Two apps have been developed for quantitation by smartphones, one for working with RGB values and the other for spectra obtained by the miniaturized spectrophotometer coupled to a smartphone.

## Introduction

Increasing demands for new devices for monitoring the environment^1,2^ and health,^3^ including security and environmental protection, at the point of the problem have contributed to the development of portable instrumentation.^4^ Portable spectroscopy is a very significant and growing discipline, and hence, the number of articles related to this topic in the web of science (WOS) database has increased considerably in the last 10 years (to more than 20 000), and 25% of these articles are from the chemistry field. However, more than 35 000 articles related to smartphones have been published, of which only 5% are related to chemistry.

For solid colorimetric devices, paper,^5^ PDMS,^6,7^ and cotton^8^ were proposed as supports. Traditionally, color is measured by the naked eye for qualitative or semiquantitative analysis^9^ or by a noninvasive technique such as lab-visible reflectance spectroscopy (from 380 to 780 nm)^9^ for quantitative analysis. However, there is a possibility of configuring cheaper portable instruments using portable components and optical fibers, including a reflection probe.^10,11^ Nowadays, smartphones are widely used by people; thus, they have become a valuable tool within the framework of in situ analysis. Guo implemented blood β-ketone monitoring by the utilization of a smartphone-powered medical dongle as a miniaturized electrochemical analyzer,^12^ while Fu et al. presented a palm-sized uric acid test powered by a smartphone using a photochemical dongle for proactive gout management.^13^ In addition, Mu et al. reported a nano-SERS chip combined with a smartphone-based Raman detector for the identification of pesticide residues.^14^ Accordingly, in the last decade, new “color instruments” became available for much lower prices as well, thanks to digital cameras and smartphones.^15,16^ A smartphone can capture images, which can be processed in order to obtain parameters related to color, such as RGB (red/green/blue) coordinates^14^ or CIElab,^15^ among others. Several works have been reported in past years in analytical chemistry, in which these color coordinates have been used as analytical signals for calibration.^15−19^ This smartphone-based colorimetric test needs illumination and image processing. However, the image quality can suffer from nonuniform and nonreproducible lighting, which negatively affects the accuracy of the measurement. To obtain good measurements, some strategies such as the use of boxes with light (LEDs)^20^ or using the light source of the phone^18^ have been proposed to eliminate the interference from ambient light. But the spectrum provides more selective information of a given analyte. In this sense, combining the integration of CMOS (complementary metal–oxide–semiconductor) camera image sensors with an optical grating and a spectrum processing technique, a portable spectrophotometer can be obtained. Some examples have been described in the literature based on the smartphone spectrophotometer design using transmission and reflective diffraction gratings.^21−23^ All of the spectrometer components can be integrated into a small block and can be attached to the phone camera. Because of the small size and easy portability, this instrument is an alternative to conventional optical spectroscopic techniques and is suitable for different applications.^24^ In terms of software, the data acquisition and processing capacity of the phone itself play an important role.^25,26^

The analytical properties of different measuring instruments have been obtained and scaled in this paper for solid chemosensors of ammonia and hydrogen sulfide as use cases (see Table S1 for the type of samples and levels of concentration).^27−29^ The smartphone (coupled or not to a miniaturized spectrometer) and portable reflectance instruments have been compared with a laboratory reflectance instrument. First, a protocol guide to perform suitable measurements using a smartphone has been established employing a set of 45 colors, and two apps were developed for quantitation by the smartphones: one for RGB values and the other for spectra obtained by the miniaturized spectrophotometer coupled to a smartphone; several smartphones were also tested. Then, a comparative study for all instruments, analytical and environmental, has been performed. Finally, as a conclusion, some rules for selecting the most appropriate instruments for obtaining the required information have been set up.

## Materials and Methods

### Reagents and Solutions

For details of the reagents and solutions used in the study, refer to the Supporting Information.

### Colorimetric Reaction on Solid Supports

For preparation of the colorimetric solid supports used in the study, refer to the Supporting Information.

### Analytical Response Measurements

Four different instruments were used: UV–Vis diffuse reflectance spectrometer (Cary 60 UV–Vis, Agilent), UV–Vis portable reflectance spectrometer (OceanOptics), smartphone, and smartphone coupled to a miniaturized spectrometer (GoSpectro, Alphanov). Two different procedures were used to obtain the RGB components: (i) nonprocessed images and (ii) processed images.^30^ For details of the instrumentation used and the measurement of the analytical response, refer to the Supporting Information.

## Results and Discussion

### Panel of Color Control and Colors of Monitoring Sensors

**Figure 1:**
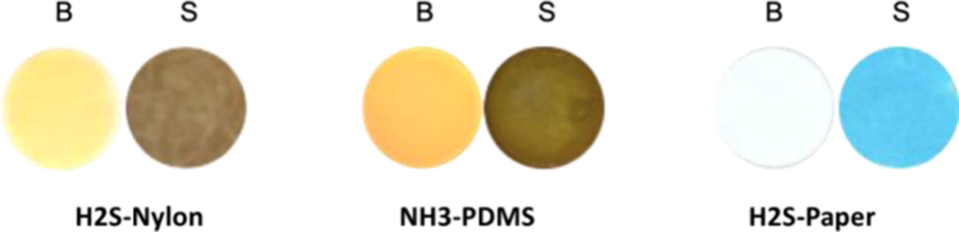
Colorimetric solid sensors using different supports (nylon, PDMS, and paper) exposed at different concentrations (B: blank and S: sample).

A color palette covering the visible color range (Figure S1) was selected as the validation set. Besides, three different solid supports (PDMS, nylon, and paper) on which chemical reactions have been carried out have been selected as cases of study. The first example was a NH_4_^+^ sensor, the second one was a paper-based sensor employed to determine H_2_S by forming methylene blue, and the last example was a plasmonic sensor of H_2_S that used AgNPs retained on a nylon membrane. In Figure 1, images of the three selected sensors (with and without exposure to the analyte) are shown; it is evident that the formed colors were different and corresponded to different spectral regions. All of these materials (paper, nylon, and PDMS) allowed light to pass through, and so, the analytical signal can be obtained using reflectance and transmittance modes.

### Responses Using a Lab Reflectance Diffuse Instrument as a Reference

Table 1 shows the characteristics evaluated for the lab equipment: some instrumental properties; portability related to size, weight, and autonomy; and cost and sustainability measured as carbon footprint according to Pla-Tolós et al.^31^ It has a spectral resolution of 1.5 nm with the wavelength in the range of 190–1100 nm. The drawbacks of this instrument are lack of portability, its cost, and a higher carbon footprint.

**Table 1:** Main Analytical Properties of the Different Types of Instruments Used

specifications	visual inspection naked eye	smartphone (digital image)	smartphone-miniaturized spectrometer	portable reflectance spectrometer	lab reflectance spectrometer
analysis type	semiquantitative	quantitative	quantitative	quantitative	quantitative
spectral resolution			5 nm	0.5 nm	1.5 nm
spectral range			380–750 nm	190–1100 nm	190–1100 nm
light source			LED or halogen bulb	halogen, vis-NIR, (HL-2000-HP-FHSA, Ocean Optics)	xenon flash lamp (80 Hz)
run time		5 s	2 s	20 s	30 s
CV (%)		<1.5	<1.5	<1	<1
light source power			10 W	20 W	9–18 W
operating system		android or iOS	android or iOS		
size			50 × 20 × 20 mm	89 × 63.3 × 32 mm	550 × 420 × 270 mm
weight			30 g (just device)	265 g (just spectrometer)	20 kg
price		300–600 €	70–1200 €	8000–10000 €	9000–12000 €
sustainability (carbon footprint)		0.0014 kg CO_2_	0.0014 kg CO_2_	0.024 kg CO_2_	0.17 kg CO_2_

**Figure 2:**
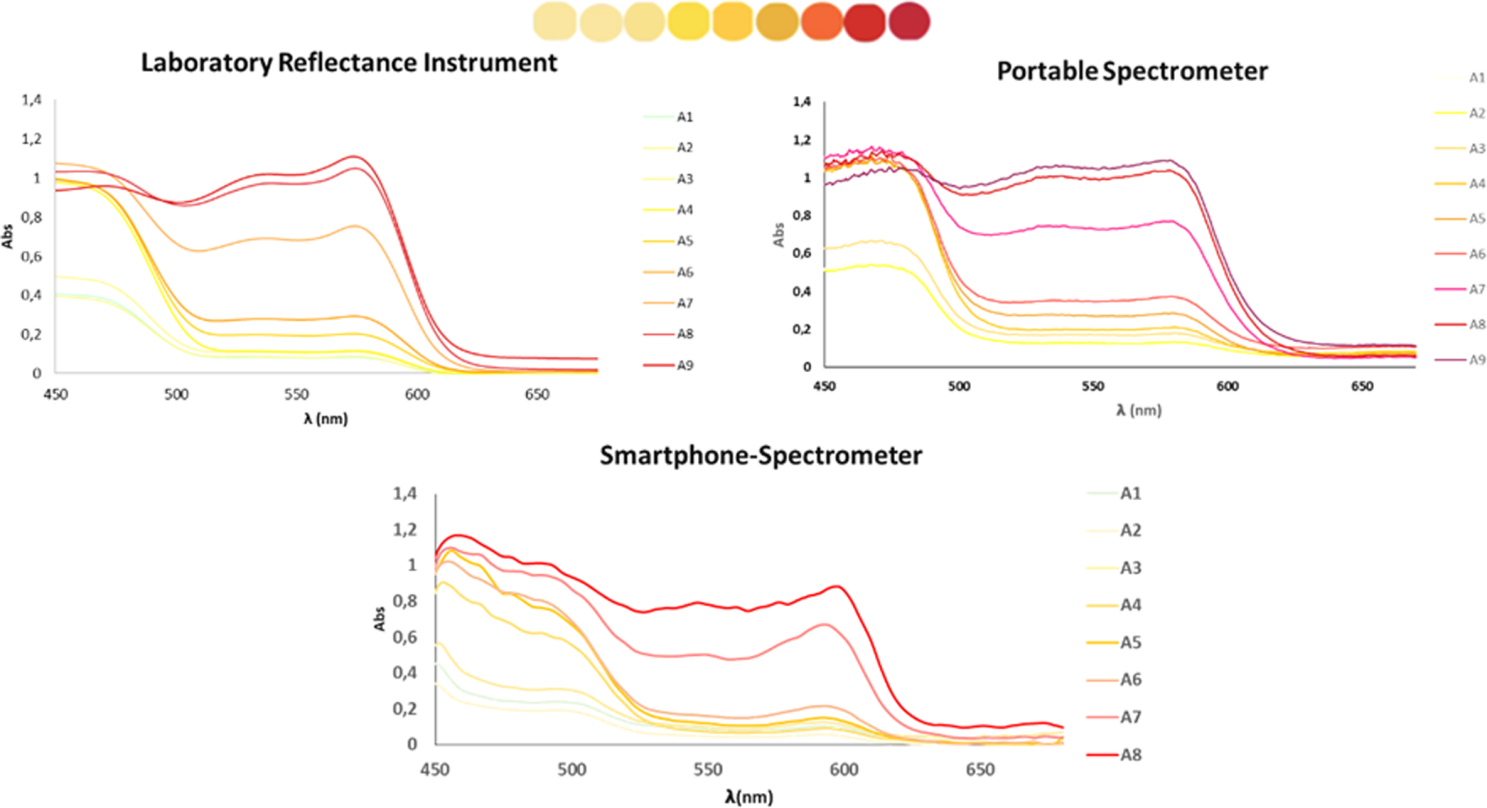
Reflectance spectra corresponding to different colors obtained using different instruments. (The absorption spectra for the smartphone-spectrometer were obtained using GoSpectro (Alpahanov) coupled to a smartphone at optimum conditions (sensor at 1 cm, halogen lamp, 20 W) in a wavelength range from 350 to 700 nm; data was obtained using the GoSpectro App developed by Alphanov. For the portable spectrometer, the absorption spectra were obtained using a UV–vis portable reflectance spectrometer (Ocean Optics), a tungsten halogen lamp of 20 W (HL-2000-HP-FHSA Light Source from Ocean Optics), and data was registered using the computer program OceanView).

To obtain quantitative analytical parameters such as S/N noise (calculated as *X̅*/s, standard deviation),^32^ the spectra of the panel of 45 colors (Figure S1) were registered, considering colors divided into five groups of nine components each. Figure 2 shows the spectra obtained by the lab instrument when different colors of the red range were measured, as an example; the S/N value obtained was 34, as shown in Table 2. The table also shows the % RSD obtained from *n* = 3 spectra registered in the same working session and in different ones (*n* = 3). As can be seen, the inter- and intraday precisions were lower than 2% for this instrument.

**Table 2:** %RSD Inter- and Intraday Deviations for Different Colors at Different Wavelengths^a^

	(%RSD)				
instrument	royal yellow (460 nm)	fire red (495 nm)	light blue (590 nm)	apple green (710 nm)	S/N
	intraday/interday	intraday/interday	intraday/interday	intraday/interday	
lab reflectance spectrometer	0.70/0.9	0.2/1.4	0.2/0.4	1.6/2.0	34
portable reflectance spectrometer	0.1/0.5	0.1/0.5	0.05/0.3	0.05/0.2	47
smartphone-spectrometer (reflectance mode)	1.5/1.6	0.4/2.2	0.8/1.2	0.3/1.6	18

a Signal-to-noise (S/R) ratio for different instruments. The S/R ratio is calculated as 5 × Standard deviation/Δsignal

### Responses with a Portable Reflectance Diffuse UV–Vis Spectrometer

The instrument used in this work consisted of a modular device constituted by a lamp, detector, fiber optic, integrating sphere of 8 mm, and a computer. As can be seen in Table 1, from the instrumental point of view, this instrument presents a very good resolution (<0.5 nm). The components can be set on a suitcase and easily transported, as their weight (taking all of the components) is around 2 kg. The price is lower than the conventional lab instrument with the diffuse reflectance accessory. Regarding sustainability, the carbon footprint is much lower than that of traditional equipment. In the case of conventional lab instruments, the measuring methodology is very well established, and the measuring conditions are usually not affected by environmental conditions.

**Figure 3:**
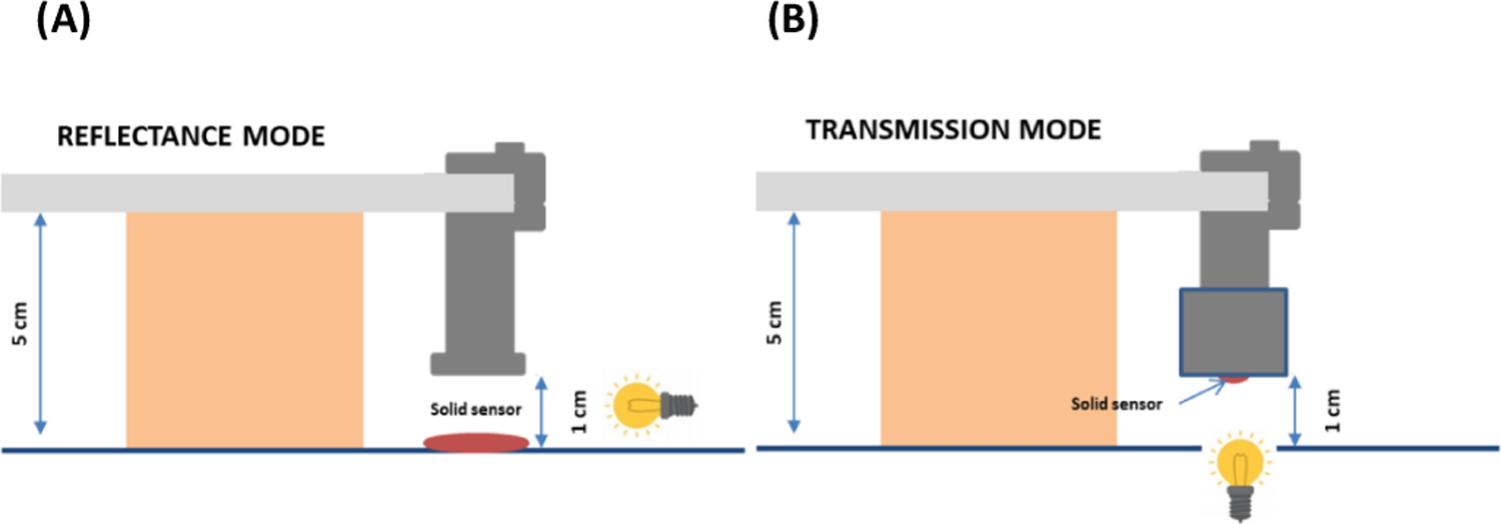
Prototype of the spectrometer adapted to a smartphone: (A) reflectance mode and (B) transmission mode.

When measurements were performed on the color palette control, the spectra obtained using this instrument are similar to those obtained by using the lab instrument (see Figure 3). The highest precision was obtained using the portable optical fiber reflectometer with lower values of % RSD. A very good S/N value of 47 was also obtained.

### Responses with a Smartphone Coupled to a Miniaturized Spectrometer: Establishing Rules for Accurate Measurements

A smartphone, coupled to a minispectrometer (GoSpectro) that uses the camera of the phone as an optical sensor, was employed. GoSpectro is an app for Android and iOS licensed by Alphanov, which allows light calibration and spectra registration. To the best of our knowledge, this is the first study where this instrument has been used for quantitative analysis. The instrumental characteristics of this device are shown in Table 1 It has a resolution of 5 nm and an optical entrance of 0.6 mm; the optical entrance parameter determines the size of the sample to be measured and the position of the device regarding the sample. It can measure in reflectance or transmittance mode (depending on whether the material is transparent or translucent), depending on whether the light is reflected off the sample or light is going through the sample. Besides, it can be connected to a fiber optic to have more precise measurements. As it has a small size and low weight, it is suitable for in situ analysis. Therefore, as can be seen in Table 1, the carbon footprint is lower than the other compared instruments. The cost of this instrument is very low. The appropriate data acquisition with the minispectrometer-smartphone devices requires controlling the parameters of the instrument (calibration) and other external parameters, such as light, sample position, or the type of mobile phone employed. This instrument is easily calibrated; however, one of the main drawbacks is that the user needs to control carefully the environmental conditions, such as light. This is a common problem for all of these types of instruments due to light affecting the color responses.^33^

Regarding the position of the instrument, it was concluded that a better way to obtain precise results was to fix the instrument (spectrometer) 90° with respect to the horizontal surface (Figure 3A), while the smartphone was placed horizontally. This position guarantees that the distance between the sample and the spectrometer device is the same all over the sample. The distance to the sample was dependent on the sample size. It was observed that the smaller the area of the sample, the closer the spectrometer device should be placed. For round samples of 1 cm diameter, the optimum distance of the spectrometer end to the sample was 1 cm, while the distance of the smartphone from the surface was 5 cm. The environmental light should also be controlled to perform the measurement. Thus, the sample spectra were registered when different lights were used (environmental light like sunlight as a natural source and fluorescent tubes (50 W) and light from a LED (5 W) or a halogen bulb (10 W) located at 10 cm from the sensor (Figure S2A)). The sunlight spectrum is more uniformly distributed and more sensitive; however, the signal precision was poor due to its dependence on the time of the day or the weather. The use of a fluorescent tube was not recommended because of its poor intensity and smaller range of lighting wavelengths. Although the LED bulb provided good results and a wide range of wavelengths, we propose the use of a halogen bulb because it provides higher sensitivity (Figure S2B). In order to perform the measurements in transmission mode, a homemade sample holder was used. It consists of a cylindrical plastic piece with a small hole that would be fixed at the extreme of the spectrometer. The sample is introduced at the base of the cylindrical piece and will be in between both the pieces (the plastic piece and the spectrometer) (Figure 3B).

Another problem of using a smartphone as an analytical instrument is the uncertainty in reproducibility across smartphone devices.^34^ The effect on the signals when using different smartphones (three different iPhone models) was tested by registering the spectra of four paper colors (red, gray, blue, and green). As can be seen in Figure S3, the shape of the spectra obtained are quite similar to each other; however, the absorbance value was dependent on the model phone. Based on these results, we can conclude that the calibration and the sample measurements should be done with the same smartphone device to obtain reproducible signals under controlled light conditions, position, and size of the sample.

By working under optimal experimental conditions, the reflectance spectra of the palette of 45 colors were registered. As can be seen (Figure 2), similar analytical signals were obtained using the reflectance instruments; however, slightly lower absorbance signals were obtained when a smartphone-spectrometer device was used. A slight shift in the maximum absorption can be observed when using the smartphone coupled to a spectrometer. These results are satisfactory, and this displacement can be explained by different spectral resolutions of different instruments. Although the values of intraday precision were higher than those given by the other instruments, these results were approximated (<10%) (Table 2). As expected, the S/N ratio was also lower than those of the other instruments compared.

**Figure 4:**
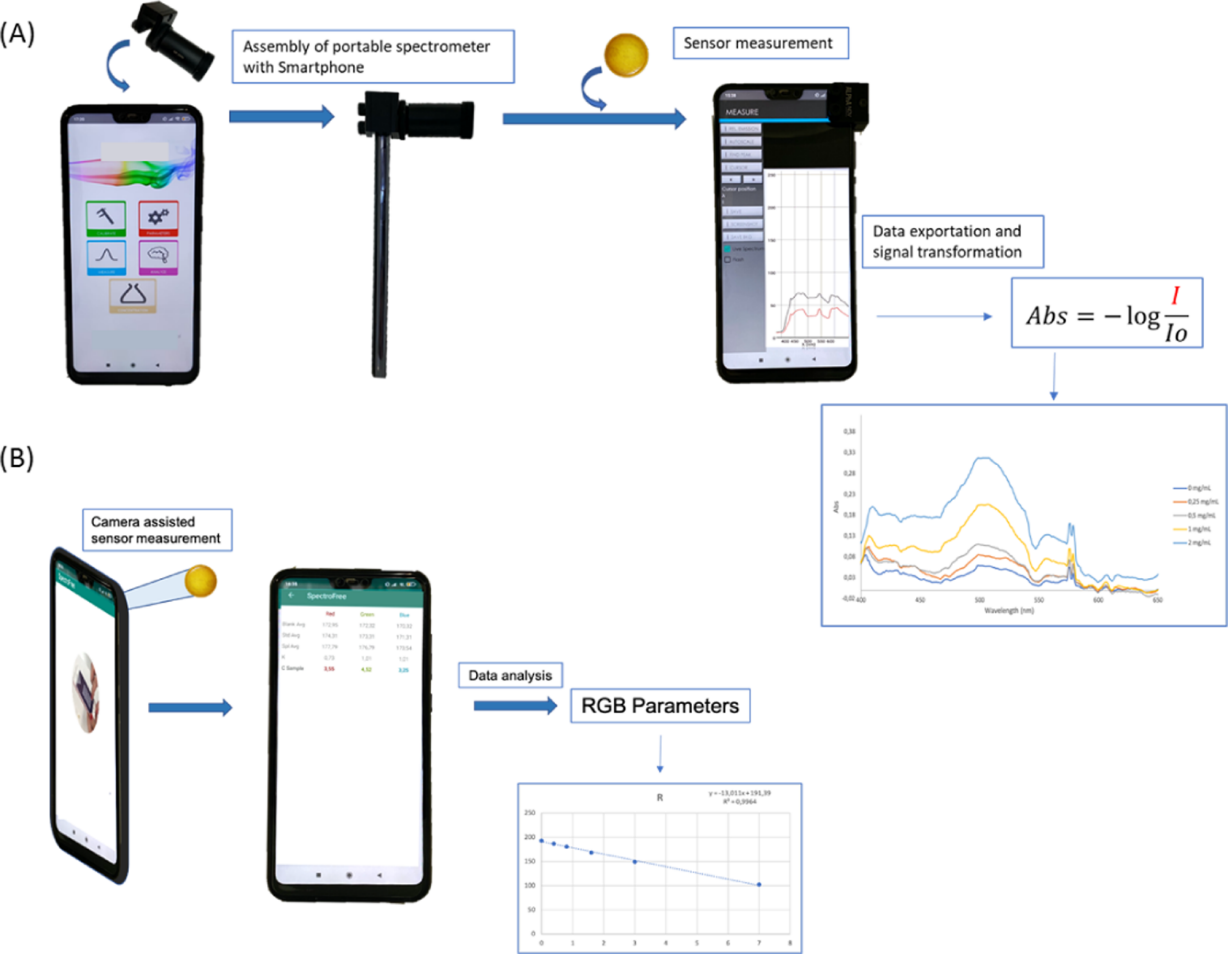
Schematic illustration of a sensor measurement using (A) a smartphone-spectrometer device and recording of the spectra. (B) Digitalized image using a smartphone camera and determination of the RGB parameters.

**Figure 5:**
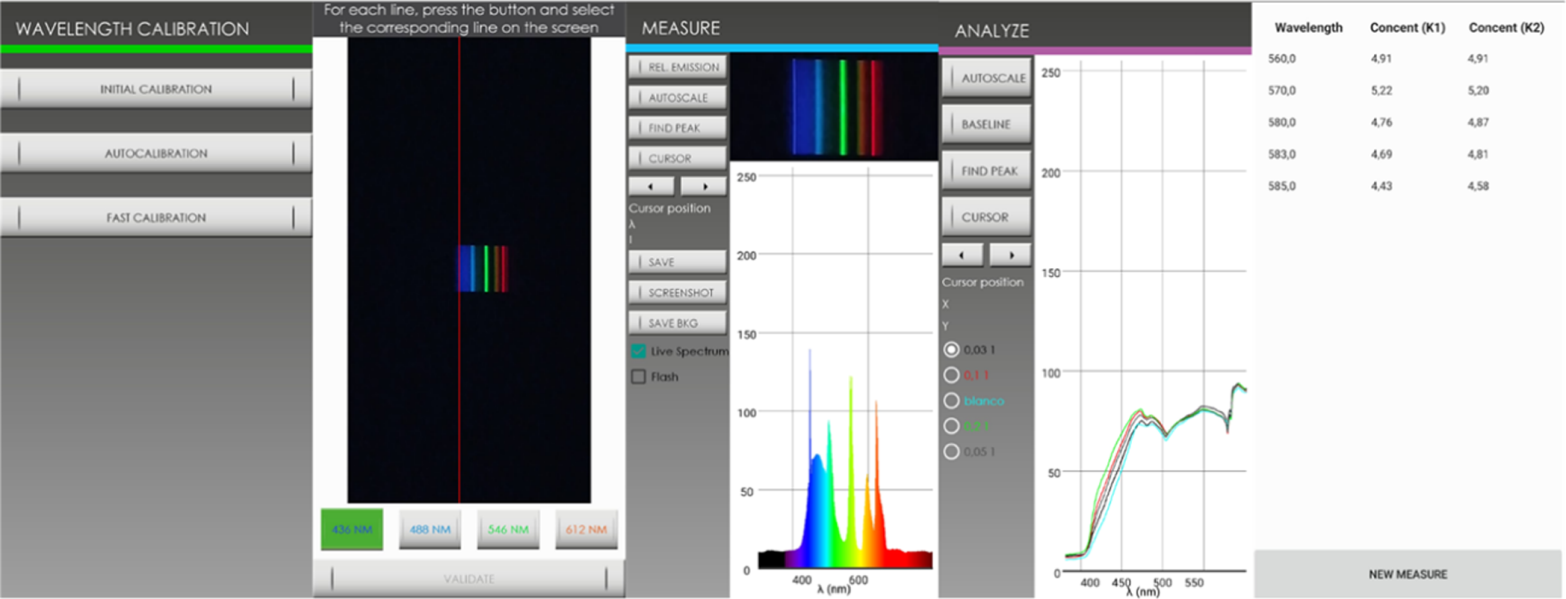
App for calculating the concentration of a sample using the spectra.

By using this instrument, the raw data obtained corresponds to the light intensity. These data should be converted to absorbance values. Therefore, we will need to register the blank signal (blank material) in order to obtain the *I*_o_ and the *I* values of the different samples. These data will be processed in order to obtain the absorbance from the transmittance using the Lambert–Beer law (Figure 4A). From the analytical point of view, there is a need to calculate the concentration using standards and their absorbance. In this case, there are two different ways to calculate it when using this prototype: (i) by exporting and processing the raw spectral data to external programs such as excel or (ii) by using an app designed for direct calculation. The app for spectrometer-smartphone software (app) has many advantages such as it is quicker, and the data can be stored and easily transferred via Wi-Fi. In this sense, a calculation option was included in the GoSpectro App from the MINTOTA research group. By clicking the calculation button, the concentration of the sample is calculated using the spectral data of the blank, two standards of known concentration, and the sample. Thus, the user will only need to measure the blank, two standards, and the sample. Calibration of one point will be used as a calibration model for each standard, in which a *K* constant = standard concentration 1/(Abs standard 1 – Abs blank sensor) will be calculated and used to calculate the concentration of the sample (*C* sample = (Abs sample – Abs blank sensor)/*K* constant) for both standards. Figure 5 shows some images of the different screens of the app. The app has the option to select up to five wavelengths for calculating the concentration and two standards for calibration.

### Response with Smartphone and RGB Color Coordinates: Establishing Rules

A smartphone can also be used to obtain digital images in order to obtain the color coordinates, and RGB is one of the most commonly used color models in image processing (Figure 4B). However, color can be converted to other models such as hue, saturation, and value (HSV); hue, saturation, and lightness (HSL); hue, saturation, and intensity (HSI); and lightness, green-red, and blue-yellow (L*a*b*). These values can be correlated with the analyte concentrations.^35^ The smartphone cameras have mostly limited control of camera parameters (e.g., exposure time, shutter speed, ISO, and color balance, and no access to raw image data), and the image processing is applied automatically and varies significantly across smartphones. These methods disturb the linearity of the pixel intensity values, which causes loss of information. On the other hand, ambient light conditions are difficult to control during imaging in uncontrolled environments.

The mobile phone can be set in the same conditions described previously for reflectance mode or using the protocol developed by Pla-Tolos et al.^36^ which used a box and artificial light for controlling the light conditions. The digitalized images obtained (JPEJ format) can be processed by external programs such as GIMP, Jimage or MATLAB in order to obtain the color components. Besides that, commercial apps, such as Color Grab* for android and color Assist for iOS, allows easily to obtain the color coordinates. These data can be used for prediction by using multivariate methods^37^ or by correlation one component^36^ or the ratio between two components with the analyte concentration.^38^

Using the conditions established previously and using a commercial app (Color Assist), the RGB components of the palette of 45 colors were obtained. The precision obtained was 4.0/3.9 for intra- and interday (*n* = 3), respectively, for a blue color as an example. Conditions such as light source, mobile and sample position, and smartphone used have to be considered to obtain reproducible signals. This problem can be reduced if images of the calibration points and the samples are taken at the same time as discussed in Analytical Response Measurements section.

**Figure 6:**
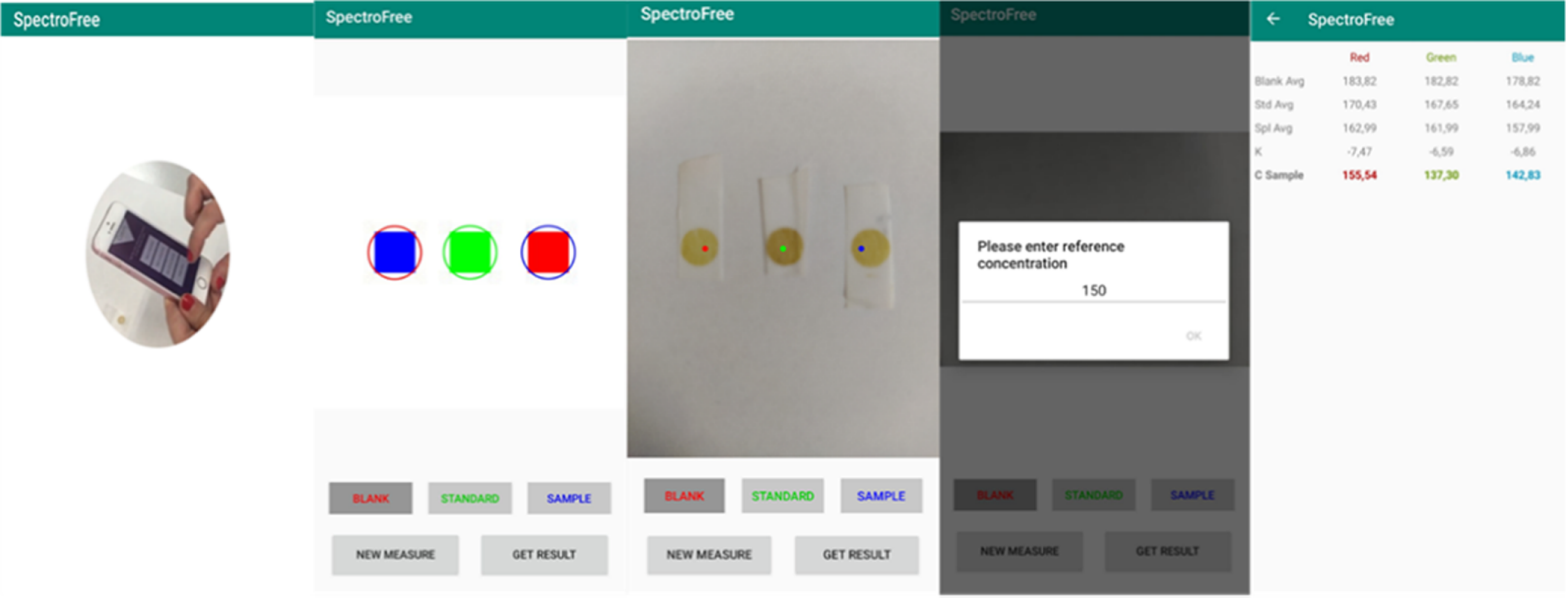
App for calculating the concentration of a sample using the RBG components.

Here, an app that allows calculating the concentration from the RGB of the image has also been developed (Figure 6). In order to determine the concentration in a sample, the user will need photos of the blank signal, a standard with a known concentration, and the sample with an unknown concentration, all at the same time. Using the calibration of one point as a calibration model, the app can calculate the sample concentration. By performing the assay under these conditions, we will guarantee that the environmental measurement conditions are equal for all of the points measured. The app will obtain the values of RGB for the blank, the standard, and the sample, and the concentration of the sample will be calculated using the three RGB components.

As can be seen in Table 1, the use of the smartphone-free spectrophotometer is one of the simpler instruments. This is a very economic option (nowadays everybody has a smartphone) and the cost to download the app is not very high.

Other aspects considered were the analytical data provided by different instruments and its conversion into analytical information. Multivariate methods were used to analyze the information provided using the spectral data and the RGB components (see the Supporting Information). The RBG univariate models present a lack of selectivity and do not have enough selectivity to establish differences between very similar colors. Small color changes cannot be detected in the analysis, as the red, green, and blue (RGB) intensity values may not be sufficient. These aforementioned concerns make smartphones limited for full applicability to quantitative analysis.

### Responses by Visual Inspection

A color change observable by the naked eye in response to the concentration of an analyte can be an indication of a problem warranting further attention. This type of methodology is very useful for qualitative or semiquantitative analysis. To estimate the concentration by visual inspection, the user will need a cart of colors corresponding to different concentrations and the sample. Although this methodology was proposed for strip reagents in the 1960s, it is still very useful.^39^ Its limitations for quantitation, selectivity, accuracy, and precision should be considered, yet it can be useful in several situations and fields.

### Solid Chemosensors as a Case Study: Scaling the Information

**Figure 7:**
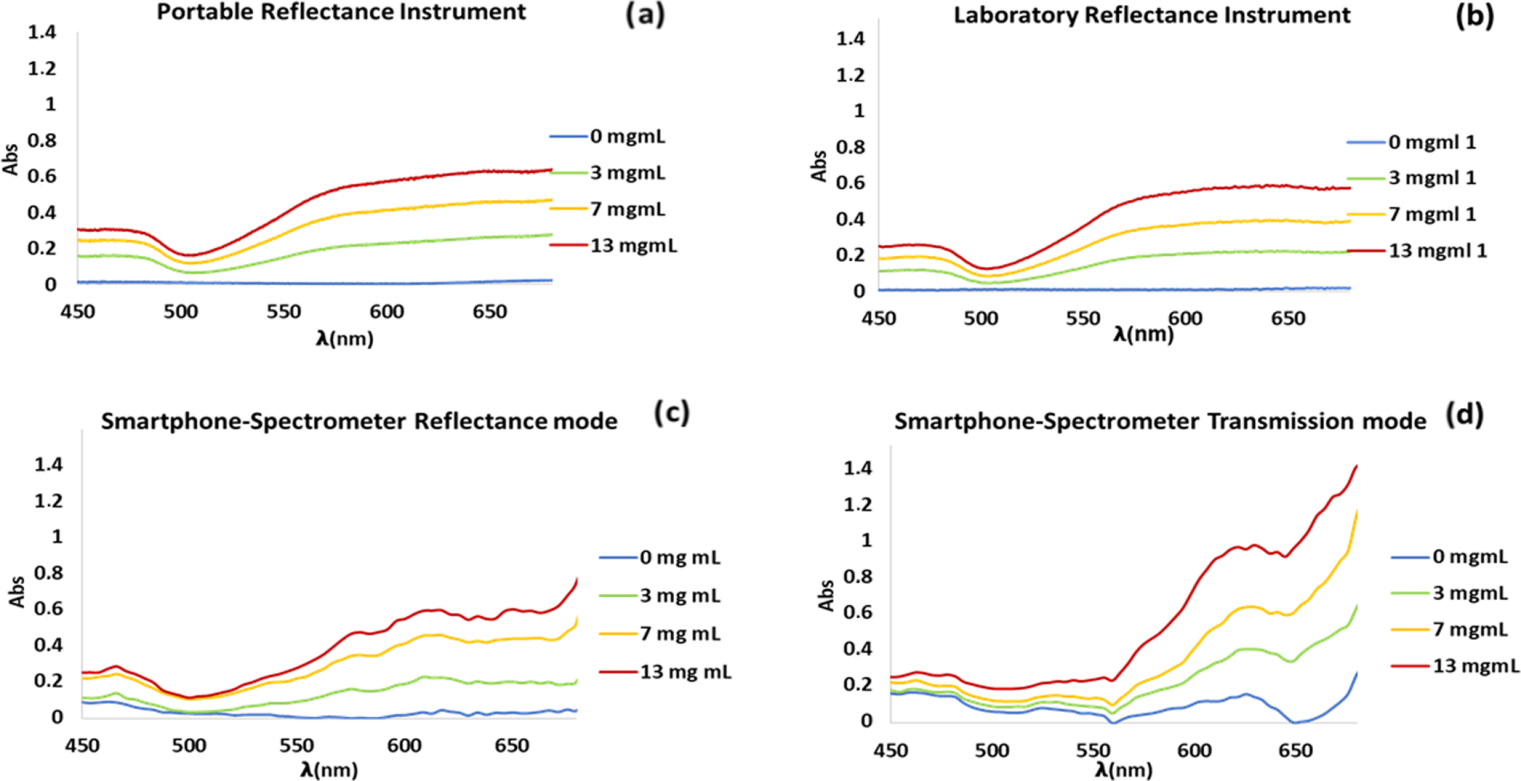
Spectra corresponding to H_2_S paper sensors using methylene blue reaction using different instruments.

The aim of this section is to establish some guidelines to select the appropriate measuring instrument for an analysis depending on the demanded information. Traditionally, this selection has been made mainly on some analytical properties but, nowadays, other ones should also be considered. Different instruments already evaluated have been used to obtain the analytical responses of the three sensors exposed at different analyte concentrations. As an example, Figure 7 shows the spectra of the H_2_S sensor based on the formation of methylene blue. The spectral shape obtained using the reflection mode was similar for all of the instruments, although the absorbance values were slightly lower for a smartphone-spectrometer. However, by measuring in the transmission mode, the absorbance values were a bit higher than those obtained in the reflectance mode. Similar results were obtained for the other sensors tested.

The figures of merit of different sensors using different instruments are shown in Table 3. For all sensors, the analyte quantitation was carried out by external calibration. The limits of detection (LODs) and limits of quantification (LOQs) were calculated as 3*s*_a_/*b*_1_ and 10*s*_a_/*b*_1_, respectively, where *s*_a_ and *b*_1_ are the standard deviations of the ordinate and the slope of the regression. The linear range, sensitivity, and precision of the methods were evaluated. The figures of merit of different sensors using portable instruments are similar to those obtained using the traditional lab instrument. Good correlations, appropriate detection, and quantification limits were obtained. In general, for the three sensors, slightly higher LODs and LOQs were obtained with a smartphone-spectrometer. On the other hand, good results were also obtained using the RGB components from the images. As can be seen, depending on the sensor, the RGB selected were different: *R* (Red) component for the NH_3_ sensor, *R* (Red) for the H_2_S sensor (in paper), and *G* (Green) for H_2_S (in nylon). In this case, the ImageJ program was used. From Table 3, it can be seen that when images were processed the analytical parameters improved slightly. In all cases, a good correlation was obtained and the analytical parameters were similar to those obtained with other instruments (lab or portable reflectance instrument).

**Table 3:** Figures of Merit of the Sensors of PDMS, Nylon, and Paper Using Different Instruments and Signals (Absorbance or RGB Components)

	intercept (*a* ± *s*_a_)	slope (ppm^–1^) (*b* ± *s*_b_)	*R*^2^	linearity range (mg/L)	LODs (mg/L)	LOQs (mg/L)
**NH_4_^+^/PDMS (λ = 600 nm)**						
diffuse reflectance spectrometer	0.334 ± 0.002	0.0204 ± 0.0004	0.999	1.0–16	0.3	1.0
portable spectrometer	0.353 ± 0.003	0.0202 ± 0.0008	0.998	1.5–16	0.4	1.5
smartphone-spectrometer	0.180 ± 0.004	0.0229 ± 0.008	0.999	1.7–16	0.5	1.7
smartphone-digital images (RGB) (*R* component)	130.6 ± 1.2	–2.79 ± 0.11	0.997	1.5–16	0.5	1.5
smartphone-digital processed images (RGB) (*R* component)	133.3 ± 1.3	–11.8 ± 0.2	0.999	1.2–16	0.4	1.2
**H**_**2**_**S/paper (λ = 650 nm)**						
diffuse reflectance spectrometer	0.018 ± 0.008	0.051 ± 0.004	0.999	1.7–7	0.5	1.7
portable spectrometer	0.04 ± 0.02	0.068 ± 0.007	0.998	3.4–7	1.02	3.4
smartphone-spectrometer	0.05 ± 0.017	0.044 ± 0.004	0.990	3.8–13	1.15	3.8
smartphone-digital images (RGB) (*R* component)	190.9 ± 1.5	–12.3 ± 0.4	0.990	3.9–7	1.2	3.9
smartphone-digital processed images (RGB) (*R* component)	191.4 ± 1.3		0.996	2.0–7	0.7	2.0
**H**_**2**_**S/nylon (λ = 500 nm)**						
diffuse reflectance spectrometer	0.2379 ± 0.0017	0.846 ± 0.008	0.999	0.02–0.4	0.006	0.02
portable spectrometer	0.284 ± 0.012	0.91 ± 0.06	0.990	0.04–0.4	0.012	0.04
smartphone-spectrometer	0.082 ± 0.006	0.57 ± 0.03	0.991	0.11–0.4	0.030	0.11
smartphone-digital images (RGB) (*G* component)	186.5 ± 2.2	–115.0 ± 10.7	0.990	0.19–0.4	0.06	0.19
smartphone-digital processed images (RGB) (*G* component)	192.8 ± 1.1	–138.4 ± 5.9	0.997	0.1–0.4	0.03	0.10

**Figure 8:**
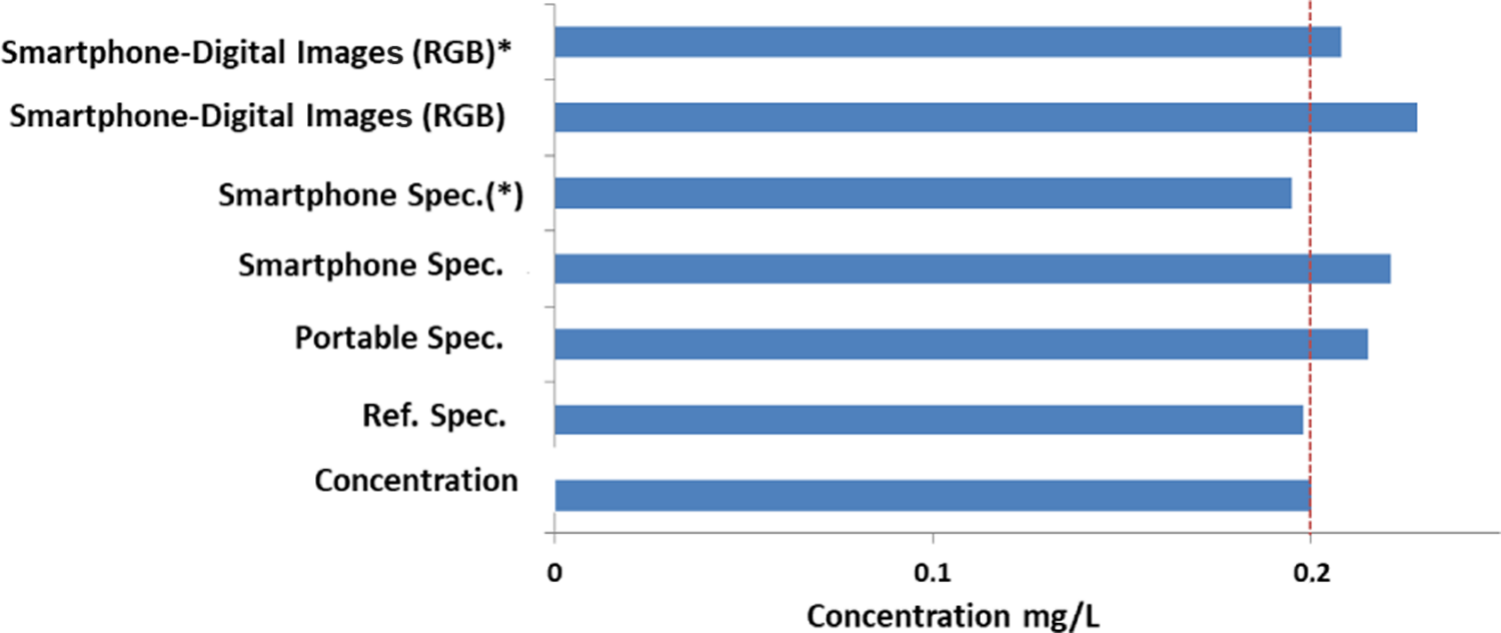
Concentration of H_2_S (H_2_S–nylon sensor with AgNPs) obtained using different instruments. (a) Smartphone-spectrometer in reflectance mode; (b) portable reflectance instrument; (c) laboratory reflectance instrument; (d) smartphone-spectrometer in transmission mode and different strategies for calculation. * Using the app designed for the smartphone-minispectra and the app for smartphone-spectrofree.

In order to evaluate the accuracy and precision, several standards of known concentration were used as samples. Table 4 shows the concentrations obtained at two different concentration levels, and the relative errors corresponding to the use of different instruments for the two sensors assayed at ppm levels. Good results were obtained for all instruments. Similar results were obtained for the H_2_S sensor of nylon at ppb levels, as can be seen in Figure 8. It is also observed that the concentrations obtained using the developed app were comparable to those calculated by the external data treatment with a smartphone-spectrometer, portable reflectance instrument, or laboratory reflectance instrument.

**Table 4:** Concentrations of H_2_S in Solution and Relative Errors Obtained Using Different Instruments

	sample 1: 4.5 mg/L	sample 2: 7 mg/L
lab reflectance diffuse spectrometer	4.68 ± 0.08	6.70 ± 0.07
	Er = 4.0%	Er = −4.3%
	RSD% = 1.7	RSD% = 1.0
portable spectrometer	4.48 ± 0.08	6.70 ± 0.04
	Er = −0.4%	Er = −4,3%
	RSD% = 1.8	RSD% = 0.6
smartphone-spectrometer^a^	4.32 ± 0.14	6.71 ± 0.05
	Er = −4.0%	Er = −4.14%
	RSD% = 3.2	RSD% = 0.7
smartphone-spectrometer^b^	4.64 ± 0.12	6.8± 0.3
	Er = 3.2%	Er = −2.8%
	RSD% = 2.4	RSD% = 3.9
smartphone-digital images (RGB)^a^ (*R* component)	4.34 ± 0.09	7.1 ± 0.2
	Er = −3.6%	Er = 1.4%
	RSD% = 2.0	RSD% = 2.8
smartphone-digital images (RGB)^b^ (*R* component)	4.6 ± 0.3	7.3 ± 0.4
	Er = 4.0%	Er = 1.2%
	RSD% = 4.8	RSD% = 2.7

a Using external data programs (Excel).

b Using GoSpectra or spectrofree calculation app.

Based on the information provided in Table S1, it can be seen that the tested analytes are important in different fields, and in some cases, the concentration levels are regulated. If analytical characteristics of smartphone methodologies are compared to others employing instruments, it can be seen that these options are the cheapest and the easiest for in situ analysis. They are also the most sustainable. The use of lab and portable reflectance instruments are always a good choice if accuracy and precise results are required or even for validation studies of the other instruments. Although the smartphone requires strict control of the environmental conditions (light source, mobile position, mobile model, sample size), the operation is very simple. A nonspecialized person can carry it out by following a protocol. The use of smartphones has the advantages of portability, computing power, memory, capability to connect to other IT systems, and the signal or captured images can be transmitted to a custom-designed app for being processed using an appropriate algorithm, which allows obtaining the concentration. Although a smartphone is the best selection for analysis in situ, it can also be used as a lab instrument if a fast analysis and/or cheap analysis is required.

## Conclusions

In this paper, the analytical information given by different types of instruments was scaled, and the main advantages and drawbacks of several instruments that can be used to measure color on the surface are discussed. Instruments such as a reflectance lab instrument, a reflectance portable instrument, and a smartphone (as an image grabber or coupled to a minispectrometer) have been compared from different points of view, such as analytical and environmental, among others. As a case study, the color developed on three solid chemosensors made of different materials, paper, PDMS, or nylon, has been tested. The figures of merit—linearity, LODs, LQDs, precision, and accuracy—obtained using lab instruments are slightly better than using a smartphone. In the case of using a smartphone, as long as the measurement conditions (light source, mobile phone position, model, sample size) are controlled, the measurements obtained will be suitable; in this sense, here some rules have been established. When we use the smartphone-spectrometer option, more precise results can be obtained using a fiber optic to capture the light. The achieved results indicate that the smartphone is a good alternative for in situ analysis or for fast and/or cheap analysis, either using the RGB coordinates from images, which can be processed in order to improve the values of coordinates, or obtaining the spectra; the latter option improves selectivity. On the other hand, the use of smartphone has the advantages of having an app to easily obtain raw data and directly transform them into concentrations, as demonstrated here, and the results can be easily stored and/or transferred and it allows one to make a quick decision for solving a given problem if necessary. On the other hand, lab instruments involve a higher carbon footprint than portable instruments and smartphones. Hence, portable instruments have been shown to be a suitable, economic, and environmentally friendly alternative for in situ analysis.

## Supporting Information Available

Reagents and solutions; colorimetric reaction on solid supports; analytical response measurements; and multivariate analysis (PDF)
