# Supplementary material for: Scaling the Analytical Information Given by Several Types of Colorimetric and Spectroscopic Instruments Including Smartphones: Rules for Their Use and Establishing Figures of Merit of Solid Chemosensors
Source: Anal Chem. 2021 Apr 5;93(15):6043–52. doi: 10.1021/acs.analchem.0c03994 (PMC9177042; doi:10.1021/acs.analchem.0c03994)
Supplement: Supplementary file 1 — ac0c03994_si_001.pdf [file ac0c03994_si_001.pdf]

Supporting Information

Scaling the analytical information given by several types of colorimetric and spectroscopic instruments including smartphones: rules for their use and stablishing figures of merit of solid chemosensors

Martínez-Aviño, Adria; Molins-Legua, Carmen*; Campíns-Falcó Pilar *

MINTOTA research group. Departament de Química Analítica, Facultat de Química, Universitat de València, Dr. Moliner 50, 46100-Burjassot, Valencia, Spain.

Corresponding authors: cmolins@uv.es; pcampins@uv.es

Table of Contents

Reagents and solutions S2

Colorimetric reaction on solid supports.. S2

Analytical response measurements S3

Multivariate Analysis S3

Table S1. Characteristics of the types of samples, legislation and concentrations.

Figure S1. Color palette used to stablish the smartphone operability.

Figure S2. A) light intensity emitted by different light sources. B) Reflectance spectra corresponding to the blue color by using different light sources.

Figure S3. Spectra obtained in reflectance mode by using different mobile phones.

Figure S4. PCA plots of the 45 colors using the A) spectra obtained by using smart-phone spectrometer, B) RGB components obtained by using a smartphone.

Figure S5. Set of colors selected as a prediction set for model calibration.

Figure S6. Spectra Cooman's plots PC1 vs PC2: a) Yellow vs red b) Yellow vs Green c) Yellow vs Blue d) Blue vs Green e) Blue vs Red f) Red vs Green.

Figure S7. RGB Cooman's plot PC1 vs PC2: a) Yellow vs Red b) Yellow vs Green c) Yellow vs Blue d) Blue vs Red e) Blue vs Green f) Red vs Green.

Reagents and solutions

Ultrapure water obtained using Nanopure II system (Barnstead, United States) was used for the preparation and dilution of all solutions. N,N-Dimethyl-p-phenylendiamine dihydrochloride was purchased from Sigma-Aldrich (Switzerland). Hydrochloric acid 37% and sodium sulfide hydrate were acquired from Scharlau (Spain). Iron (III) chloride hexahydrate was provided from Probus (Spain). Glycerol was obtained from Sigma-Aldrich (United States). Grade 41 whatman filter papers were used as a support. AgNPs (20 nm) dispersion 0.02 mg/ml in aqueous buffer solution (sodium citrate) from Sigma-Aldrich (USA). Orthophosphoric acid 85% and Sodium sulphide hydrate were purchased from Scharlau (Australia). Glycerol was obtained from Sigma-Aldrich (United States). Nylon membranes (d=0,22 µm) provided by Tecknochroma (Barcelona, Spain). PDMS silicone elastomer and curing agent were purchased from Sylgard 184. 1,2-Naphtoquinone-4-sulfonate (NQS), Tetraethyl orthosilicate and SiO_2 Nanoparticles were obtained from Sigma-Aldrich (United States). Ammonium chloride and sodium carbonate were provided by Probus (Spain).

Colorimetric reaction on solid supports

Hydrogen sulfide paper based sensors: Each filter paper was impregnated with 50 µL of a 1:1:0.1 mixture of 0.25 M of $FeCl_3$, 0.28 M of N,N-Dimethyl-p-phenylenediamine and glycerol.[1] After 15 min of vacuum drying, the sensor was exposed to a generated hydrogen sulfide atmosphere. The sensor was left to react for 30 min of sampling and then washed with 5 mL of water to remove the excess of reagent and seeing the blue color.

Hydrogen sulfide nylon-based sensors:_Nylon membranes (0.22 µm porous size) with trapped AgNPs (20 nm particle size) were used to detect H_2S compound.[2] The sensors were stored in dark at 4 ºC. For the analytical reaction, the sensors were exposed to different H_2S concentrations changing their color.

Ammonia PDMS based sensor: PDMS doped with NQS sensors were synthetized according to N. Jornet-Martínez et al.[3] and then placed in a reaction vial containing 1mL of Na_2CO_3 buffer

solution (pH=11) and 1 mL of NH_4^+. The vial was closed and heated to 100ºC for 10 min. In presence of ammonia, the sensors changed their color.

Analytical response measurements

Four different instruments were used: UV-Vis Diffuse Reflectance Spectrometer (Cary 60 UV-Vis, Agilent), UV-Vis Portable Reflectance Spectrometer (OceanOptics), smartphone and smartphone-coupled to a miniaturized spectrometer (GoSpectro, Alphanov). The absorption spectra in the last case were registered at the optimum conditions (sensor at 1 cm, halogen lamp 20 watts) and the wavelengths used for quantitation were 650, 500 and 600 nm for H_2S paper sensor, H_2S nylon sensor and NH_4^+ -PDMS sensor, respectively. Three different smartphones were used (iPhone 5S, iPhone 6 plus, iPhone SE). Smartphone-camera combined with free colour treatment computer program (ImageJ) was used to obtain the RGB (red, green, blue) components (numerical colour values). Two apps were developed for quantitation by the smartphones, one for RGB values and the other for spectra.

Multivariate Analysis

In **Figure S5** a comparison is performed for the Unsupervised (Principal Component Analysis-PCA) plots corresponding to the measurement of 45 different colors by using the absorbance values at different wavelengths obtained by the lab instrument (ranged from 400 to 900 nm) and the intensity, brightness and RGB values obtained by using the ImageJ program. Supervised (soft independent modeling of class analogy -SIMCA) models were also employed.

For both models PC1 and PC2, explained almost all the variance, being PC1 70% and PC2 30% and PC1 72% and PC2 23% for spectra data and image parameters respectively. In both cases several clusters could be observed, distributed depending on the color. The primary colors, green, blue and red were clearly distrusted forming clusters. The mixtures colors (yellow, grey, or brown) were located between the primary colors employed. Better distribution was observed

by using spectral data. When SIMCA was applied as classification model, the results obtained by using the data from images were similar to that obtained by using the spectral data. These models were used to predict four colors, the use of RGB components provided less accuracy in the prediction models, being the results from the spectra data more satisfactory in the classification analysis. There is some limitation for distinguish similar colors using the color coordinates.

The SIMCA class models were constructed based on 38 colors from the color palette (Figure S1). Four colors were used as a prediction set (**Figure S6**) for model validation. The colors were classified using 6 category variables, Blue, Green, Red, Brown, Grey and Yellow.

In order to obtain more information from the classificatory supervised models, Cooman's plots were represented for both data sets (Spectra data and RGB data).
Cooman's plots show class distances for two classes against each other in a scatter plot**. Figure S6** shows the successfully classification when using spectra data, as the samples lay well beyond the 95% confidence level in their respective areas.

Meanwhile, when RGB data is used, a less accurate classification is performed as Sample 1 fits well in the lower left-hand area in all plots conforming with all the models as seen in **Figure S7**.

The differences in prediction accuracy are caused by the quantity of information provided in each data set. While the information in RGB model is given by 5 variables, the Spectra model gives rise to 1200 variables yielding a more accurate classification as it is observed in **Figure S6 and S7**.

Table S1. Characteristics of the types of samples, legislation and concentrations.

TYPE OF SAMPLE	*Analyte*	*LEGISLATION*	*MAXIMUN CONCENTRATION ALLOWED (mg/L)*
**Drinking water**	**NH_4^+**	**Drinking Water Directive (98/83/EC)**	**0.5 mg/l**
**Superficial water for consumption**	**NH_4^+**	**Directive (98/83/EC)**	**0.5 mg/l**
**Superficial water (rivers)**	**NH_4^+**	**R.D. 817/2015 (Spain) Directive 2000/60/CE**	**<1 mg/L**
**Aquarium**	**NH_4^+**	**Non legislated**	**-**
**Drinking water**	**H_2S**	**Non legislated**	**-**
**Air**	**H_2S**	**Cal/OSHA PEL**	**10 ppm; (ST) 15 ppm (C) 50 ppm**
		**The Directive 2009/161/EU**	**8 hour TWA: 5 ppm (7 mg/m3) STEL (15 mins): 10 ppm (14 mg/m3)**
**Breath samples**	**H_2S**	**Non legislated**	**0,045-0,1 mg/L**

Directive 98/83/CE, modified in 2003,2009, and 2015 ; Cal/OSHA PEL- California Division of Occupational Safety and Health (Cal/OSHA) Permissible Exposure Limits (PELs)

Figure S1. Color palette used to stablish the smartphone operability.

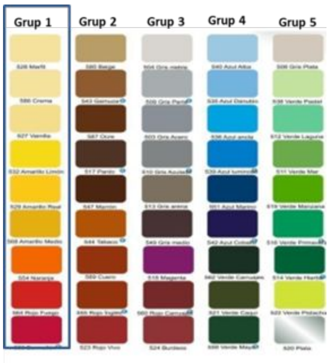

Figure S2. A) light intensity emitted by different light sources. **B)** Reflectance spectra corresponding to the blue color by using different light sources.

A) **B)**

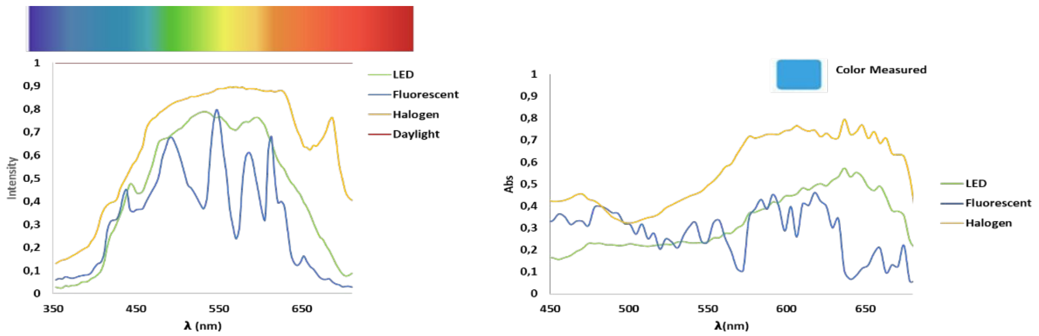

Figure S3. Spectra obtained in reflectance mode by using different mobile phones.

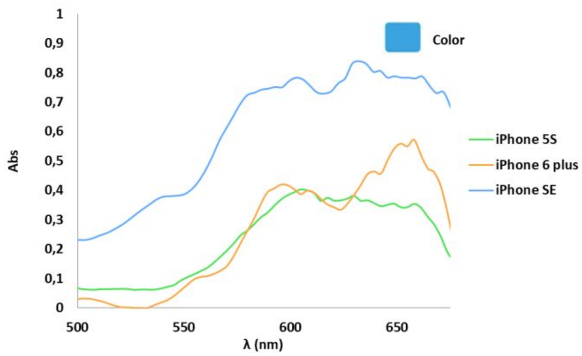

Figure S4. PCA plots of the 45 colors using the **A)** spectra obtained by using smart-phone spectrometer, **B)** RGB components obtained by using a smartphone.

A) **B)**

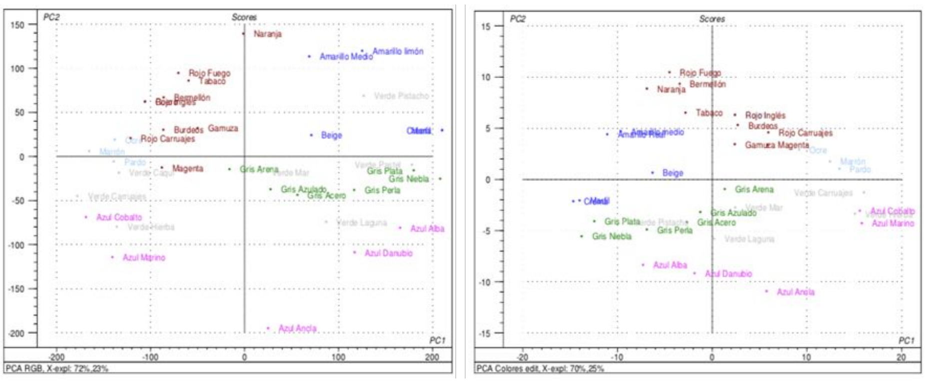

Figure S5. Set of colors selected as a prediction set for model calibration.

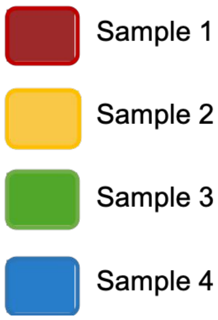

Figure S6. Spectra Cooman's plots PC1 vs PC2: **a)** Yellow vs red **b)** Yellow vs Green **c)** Yellow vs Blue **d)** Blue vs Green **e)** Blue vs Red **f)** Red vs Green.

a)

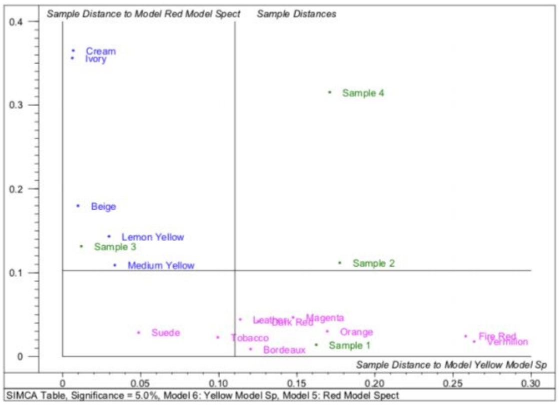

b)

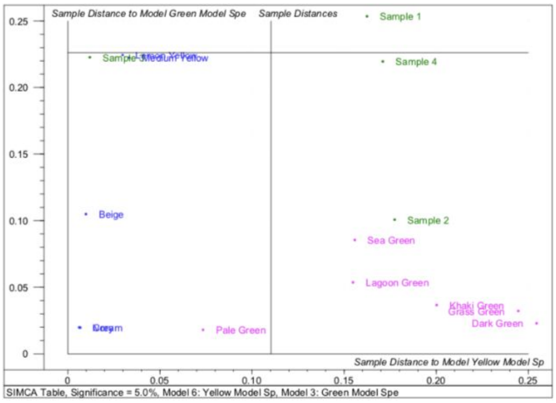

c)

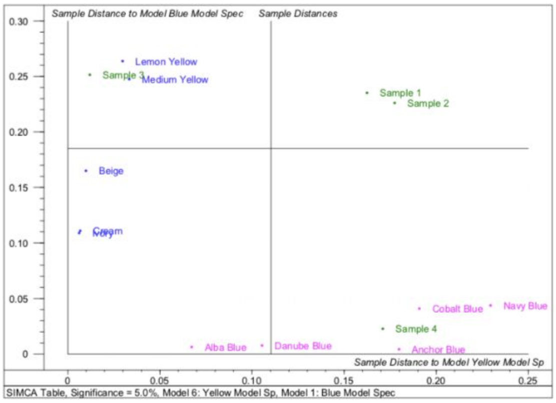

d)

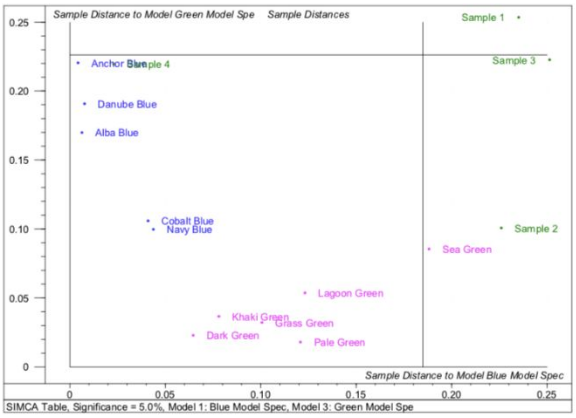

e)

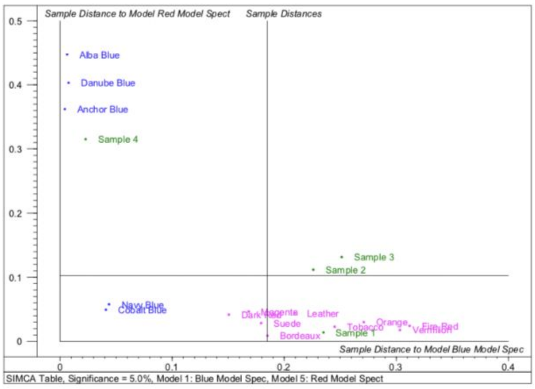

f)

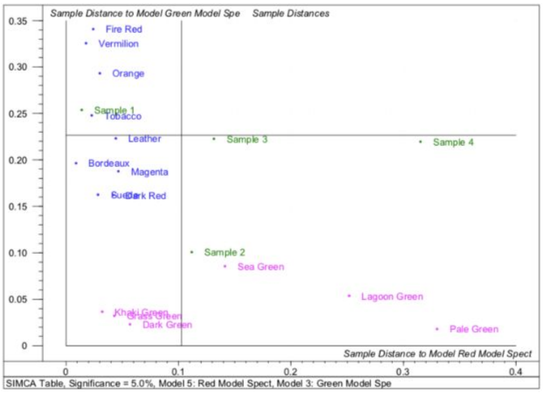

Figure S7. RGB Cooman's plot PC1 vs PC2: **a)** Yellow vs Red **b)** Yellow vs Green **c)** Yellow vs Blue **d)** Blue vs Red **e)** Blue vs Green **f)** Red vs Green.

a)

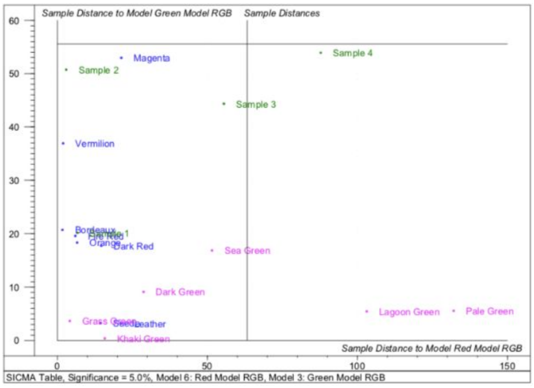

b)

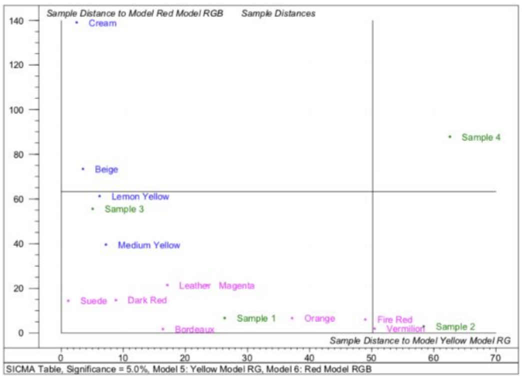

c)

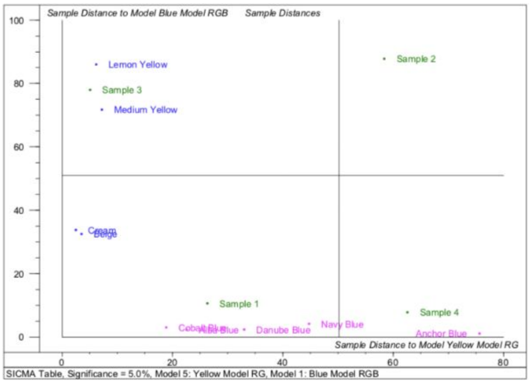

d)

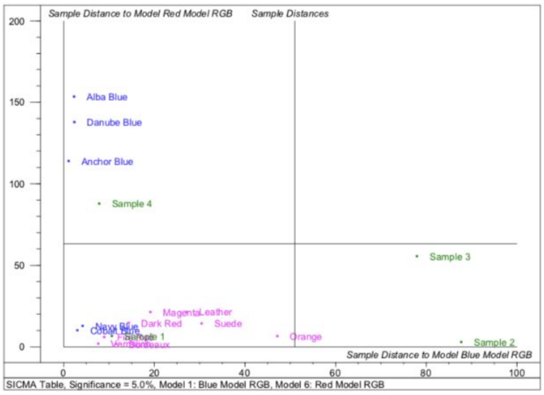

e)

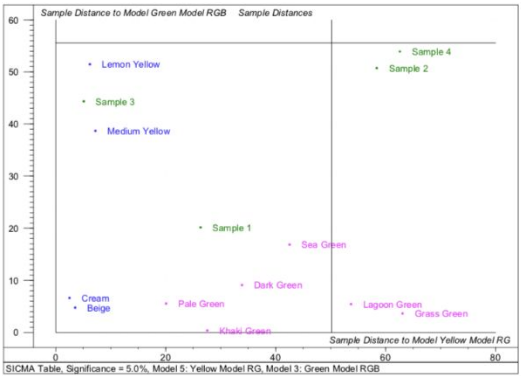

f)

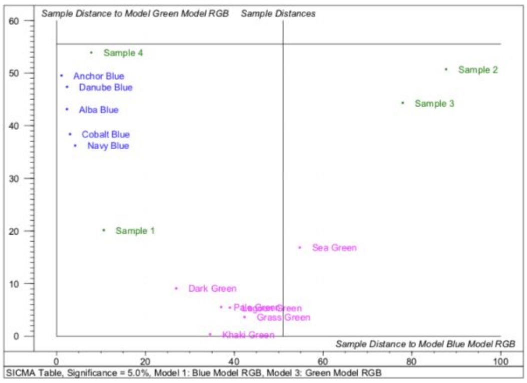

References

1. Pla-Tolós, J.; Moliner-Martínez, Y.; Verdú-Andrés, J.; Casanova Chafer, J.; Molins Legua, C.; Campins Falcó, P. New optical paper sensor for in situ measurement of hydrogen sulphide in waters and atmospheres, *Talanta* **2016**, *157-157*, 79-86.
2. Jornet-Martínez, N., Hakobyan, L.; Argente-García, A.; Molins-Legua, C.; Campíns-Falcó, P. Nylon Supported Plasmonic Assay Based on the Aggregation of Silver Nanoparticles: In-situ Determination of Hydrogen Sulphide like Compounds in eath Samples as a Proof of Concept, *ACS Sens*. **2019**, *4*, 2164-2172.
3. Jornet-Martínez, N.; Moliner-Martínez, Y.; Herráez-Hernández, R.; Molins-Legua, C.; Verdú-Andrés, J.; Campíns-Falcó, P. Designing solid optical sensors for in situ passive discrimination of volatile amines based on a new one-step hydrophilic PDMS preparation, *Sens. Actuators B: Chem*. **2016**, *223*, 333-342.
